# Influence of beef genotypes on animal performance, carcass traits, meat quality, and sensory characteristics in grazing or feedlot-finished steers

**DOI:** 10.1093/tas/txab214

**Published:** 2021-09-21

**Authors:** Isabella C F Maciel, J P Schweihofer, J I Fenton, J Hodbod, M G S McKendree, K Cassida, J E Rowntree

**Affiliations:** 1 Department of Animal Science, Michigan State University, East Lansing, MI 48824, USA; 2 Michigan State University Extension, Port Huron, MI 48060, USA; 3 Department of Food Science and Human Nutrition, East Lansing, MI 48824, USA; 4 Department of Community Sustainability, Michigan State University, East Lansing, MI 48824, USA; 5 Department of Agricultural, Food, and Resource Economics, Michigan State University, East Lansing, MI 48824, USA; 6 Department of Plant, Soil and Microbial Sciences, Michigan State University, East Lansing, MI 48824, USA

**Keywords:** beef cattle, crossbreeding, finishing systems, grass-fed beef, grazing

## Abstract

A 2-yr study was conducted to evaluate the effects of beef genotypes and feeding systems on performance, carcass traits, meat quality, and sensory attributes. A 2×2 factorial experiment was used to randomly allocate 60 steers in year 1 (YR1) and 44 steers in year 2 (YR2). The two beef genotypes evaluated were Red Angus (RA), and RA x Akaushi (AK) crossbreed. The steers were allotted to two finishing feeding systems: grazing, a multi-species forage mixture (GRASS) and feedlot finishing, conventional total mixed ration (GRAIN). All steers were slaughtered on the same day, at 26 and 18 mo of age (GRASS and GRAIN, respectively), and carcass data were collected 48 h postmortem. Growth and slaughter characteristics were significantly impacted by the finishing system (*P* < 0.01), with the best results presented by GRAIN. Beef genotype affected dressing percent (*P* < 0.01), ribeye area (*P* = 0.04), and marbling score (*P* = 0.01). The AK steers had a tendency (*P* = 0.09) for lower total gain; however, carcass quality scores were greater compared to RA. There was a genotype by system interaction for USDA yield grade (*P* < 0.01), where it was lower in GRASS compared to GRAIN in both genotypes, and no difference was observed between the two genotypes for any GRASS or GRAIN systems. There was no difference in meat quality or sensory attributes (*P* > 0.10) between the two genotypes, except that steaks from AK tended to be juicier than RA (*P* = 0.06). Thawing loss and color variables were impacted by the finishing system (*P* < 0.01). L* (lightness) and hue angle presented greater values while a* (redness), b* (yellowness), and chroma presented lower values in GRAIN compared to GRASS. Sensory attributes were scored better in GRAIN than GRASS beef (*P* < 0.01). There was a genotype by system interaction for flavor (*P* = 0.02), where beef from RA had a lower flavor rating in GRASS than in GRAIN, and no difference was observed for AK. Within each system, no difference was observed for flavor between RA and AK. Beef from steers in GRASS had greater (*P* < 0.01) WBSF than those from GRAIN. These results indicate that steers from GRAIN had superior performance and carcass merit and that AK enhanced these traits to a greater degree compared to RA. Furthermore, the beef finishing system had a marked impact on the steaks’ sensory attributes and consumer acceptability. The favorable results for texture and juiciness in GRAIN, which likely impacted overall acceptability, may be related to high marbling.

## INTRODUCTION

The National Beef Quality Audit has documented the carcass characteristics of cattle harvested in the United States over the past few decades. Researchers have observed that average hot carcass weight and the longissimus muscle area continued to increase, as well as marbling scores and quality grade. In addition, the number of carcasses that received discounts decreased, which suggests an enhancement in the consistency of produced and marketed carcasses (Boleman et al, 1998; McKenna et al, 2002; Garcia et al., 2008; Moore et al., 2012). Continued improvement in the genetics of the cattle population, combined with nutritional changes, is the main factor to improve carcass consistency, even if this is slow to produce change (Gonzalez and Phelps, 2018).

There is a need for a better understanding of the interactions among nutrition and management, finishing performance, carcass traits, and consumer acceptability, especially with regard to grass-fed beef (Neel et al., 2007). Grass-finished beef has higher proportions of beneficial nutrients to human health compared to conventionally finished beef (Duckett et al., 2007; Duckett et al., 2013; Chail et al., 2016). A survey of commercially grass-fed beef (Bronkema et al., 2019) indicates that beef from cattle finished fed solely on fresh forages had a lower *n*-6:*n*-3 ratio and greater α-tocopherol and β-carotene contents than those finished on harvested feeds. Besides the health benefits, grass-fed beef has been perceived in recent years as an environmentally friendly alternative to grain-fed beef. Topics such as sustainability and environmental compatibility are becoming increasingly common and have emerged as central elements in recent marketing campaigns (Drouillard, 2018). Some studies have shown that when including soil C changes, the overall CO_2_ equivalent can decrease considerably in grazing systems (Pelletier et al., 2010; Stanley et al., 2018; Mosier et al., 2021), and this decrease is attributed to the quality and productivity of the pastures, potentially increasing soil carbon sequestration, and thereby negating atmospheric emissions.

Diet is the main factor affecting the carcass weight and quality in young finishing cattle (Vahmani et al., 2015). Grass-fed systems are more susceptible to adverse climatic variations and grazing animals consume lower quality diets than those in confinement operations. This low-quality diet drives reduced animal performance (Thompson and Rowntree, 2020). However, diverse forage allows animals to select the plants and plant parts they consume, resulting in greater performance when animals are grazed in lower stocking densities (MacAdam and Villalba, 2015; Thompson and Rowntree, 2020). Multi-species forage mixtures are becoming increasingly popular in forage production. Aside from meeting nutrient requirements of finishing cattle, high-energy mixed-species forage accumulates evenly, is highly palatable, and sustains vegetative biomass later in the growing season in comparison to perennial pastures (Bonin and Tracy, 2012; Villalobos and Brummer, 2017; Bainard et al., 2020). Thus, these forages are able to support finishing growth, marbling and fat deposition, as well as carcass quality grade.

Crossbreeding is a frequent practice to produce calves for fattening and finishing, with the additional advantage of obtaining hybrid vigor (Gregory and Cundiff, 1980). Many studies have shown that genetic variation can increase carcass grade and meat quality (Papaleo Mazzucco et al., 2016; Mwangi et al., 2019) and alter fatty acid concentrations in beef tissues (Malau-Aduli et al., 2000; Pitchford et al., 2002; Ekine-Dzivenu et al., 2014), indicating that both quality grade and beef fatty acid profile can be improved through genetic selection. Variations in beef fat deposition due to genetic differences between cattle have been reported; Japanese breeds produce carcasses that have higher percentages of MUFA than Angus steers (Oka et al., 2002; Chung et al., 2006). Furthermore, a genetic correlation of 0.91 between marbling score and muscle lipid content was reported by Hocquette et al. (2010).

The acceptance index of beef quality is measured in relation to color, price, marbling level, subcutaneous and intramuscular fat content, and cut thickness. Furthermore, tenderness, juiciness, and flavor have been considered the three most important factors that determine the palatability of beef (Gonzalez and Phelps, 2018; Mwangi et al., 2019). However, there is very little information concerning the meat quality and the sensory attributes, especially how beef genotypes and diet can improve the quality and consistency of grass-finished beef.

Our treatment combinations allowed for a high fat (Akaushi) versus moderate fat (Angus) genotypes, in either a grass or grain framework. We hypothesized that Akaushi would have a higher carcass grade and better meat quality compared to Angus in grass finishing systems. The objectives of this study were to evaluate the influence of beef genotype on the performance, carcass characteristics, meat quality, and sensory attributes of cattle finished on either grass or grain systems. This information sets the stage for the impact of beef genotype and finishing systems on fatty acids composition that will be explored in future publications.

## MATERIALS AND METHODS

The Michigan State University Institutional Animal Care and Use Committee approved the research protocols for the use of animals and procedures (IACUC #201800155).

### Experimental Design

**Figure 1.**
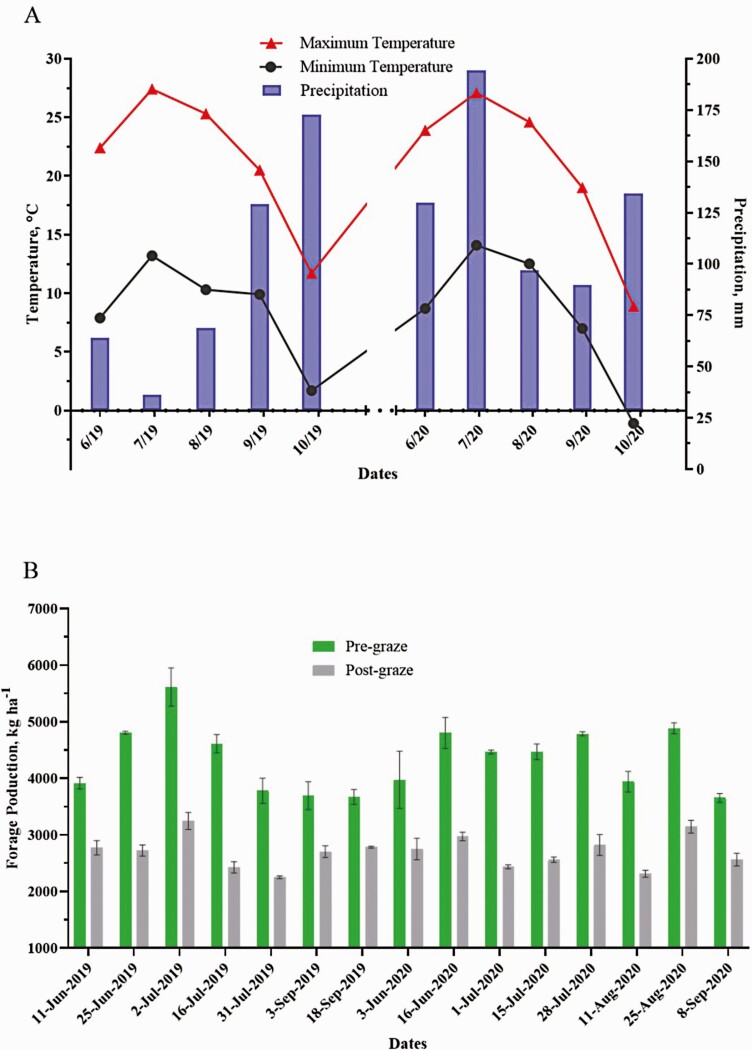
Chatham weather in 2019 (YR1) and 2020 (YR2) (A) and forage mass in kg ha^−1^ of pre- and post-graze (B) over the experiment period.

The study was conducted at the Michigan State University Upper Peninsula Research and Extension Center, UPREC (latitude 46°20′N, longitude 86°55′W, elevation 271 m) located in Chatham, MI. The trial consisted of two consecutive years (2019 and 2020). Weather data were collected from the weather station located at UPREC (Figure 1A).

A 2×2 factorial experiment was used to randomly allocate 60 steers (14–20 mo old) in 2019 (year 1, YR1) and 44 steers (14-20 months old) in 2020 (year 2, YR2). Two beef genotypes, Red Angus (RA) and RA x Akaushi crossbred (AK), were equally assigned to one of two finishing systems; a mixed-species pasture forage (**GRAS**S) or a total mixed feedlot ration (GRAIN), in three groups. For each genotype in each finishing system, animals were stratified randomly and assigned to one of the three groups.

### Animals

Each year, steers assigned to the GRASS system were selected from a herd at the Michigan State University Upper Peninsula Research and Extension Center, while steers assigned to the GRAIN system were selected from a herd at the Michigan State University Lake City AgBio Research Center (latitude: 44°18′N, longitude: 85°11′W; elevation 377 m). The GRASS steers were 6 mo older (fall born) than the GRAIN steers (spring born). The ages of the cattle for the trial were staggered such that with the extra age, the GRASS cattle would be 24–26 mo at slaughter while the GRAIN cattle would be 16–18 mo at slaughter.

In year 1, 15 RA (440.7 ± 20.6 kg) and 15 AK (438.5 ± 19.7 kg) were assigned for GRASS, and 15 RA (466.5 ± 36.7 kg) and 15 AK (473.0 ± 36.6 kg) were assigned for GRAIN. In each finishing treatment, three groups contained 5 RA and 5 AK steers. Therefore, each finishing treatment had three groups of 10 steers representing RA and AK, totaling 30 steers. The trial started on June 11, 2019, for GRASS and on June 12, 2019, for GRAIN and ended on September 27, 2019.

In year 2, due to a low number of male births, we had less steers available for the GRAIN system. Thus, 15 RA (430.6 ± 20.3 kg) and 15 AK (437.0 ± 19.0 kg) were assigned for GRASS, and 7 RA (479.8 ± 30.0 kg) and 7 AK (490.8 ± 61.9 kg) were assigned for GRAIN. For GRASS, three groups contained 5 RA and 5 AK steers, totaling 10 steers representing RA and AK. For GRAIN, two groups were established—one contained 4 RA and 4 AK, and the other contained 3 RA and 3 AK, totaling 14 steers. The trial started on June 3, 2020, and it ended on October 2, 2020.

### Grazing

Grazing consisted of an established mixed forage, which was planted in the summer of 2017 and was grazed during the summer of 2018. Animals selected for the GRASS system were rotationally grazed on mixed legumes and grass pastures for 2 wk before the start of the trial in each year.

Pastures were seeded at a rate of 19.3 kg ha^−1^, corresponding to meadow fescue (*Fetusca pratensis* (Huds.) P. Beauv.), birdsfoot trefoil (*Lotus corniculatus* L.), alfalfa (*Medicago sativa* L.), orchardgrass (*Dactylis glomerata* L.), red clover (*Trifolium pratense* L.), timothy grass (*Phleum pretense*), forage chicory (*Cichorium intybus*), and white clover (*Trifolium repens* L.).

The total area of 14 hectare was divided into eight paddocks and then subdivided into sub-paddocks. The stipulated grazing area was approximately 0.2 ha per group per day. The steers were rotationally moved 5 times per week to new sub-paddocks from Monday to Friday. On the weekends, the delimited area was calculated to allow enough forage. Stocking rates were adjusted when necessary to guarantee the forage on offer always exceeded the requirements of the livestock, leaving enough residual forage.

The experimental grazing period lasted 80 d in YR1 and 121 d in YR2. In year 1, between August 6 and September 3, 2019 (28 d), animals were removed due to low forage quantity and managed together in a mixed legume and grass pasture until grazing could continue in the experimental area. These 28 d were not included in the animal performance data. Shrunk body weights were obtained from each animal before removal and after return to pasture. Steers had ad libitum access to fresh water and free choice of mineral and vitamin block supplements (Prince Corporation, Marshfield, WI) during grazing period.

To determine forage biomass, forages were randomly measured 30 times every 2 wk in each sub-paddock using the plate meter method, in pre- and post-grazing areas. At the beginning of the growing season and then 2 mo later, ten additional plate meter readings were recorded in each sub-paddock, and the sampled area was hand clipped to ground level. All clippings were weighed, dried at 55 °C in a forced-air oven for 72 h, and weighed again to calculate dry matter (DM) content. A regression line was fitted twice each year and applied to the next plate meter readings to estimate dry forage mass. Pre-grazing biomass samples were collected immediately before the steers were allowed access to fresh forage; post-grazing residual biomasses were collected after the steers were moved. Pre- and post-grazing forage samples were collected every 2 wk by randomly clipping three 0.25-m^2^ quadrats to a 5-cm stubble using Gardena 8803 battery-operated harvest shears (Ulm, Germany) in each sub-paddock. Samples were dried at 55 °C in a forced-air oven for 72 h and ground with a Wiley mill (1-mm screen; Arthur H. Thomas, Philadelphia, PA). Wet and dry weights were recorded. Approximately 500 g of samples were composited by group and underwent subsequent chemical analyses.

Botanical composition was determined monthly in all pasture areas using the dry-rank-weight method described by Mannetje and Haydock (1963) by two trained observers. For each paddock of approximately 1.75 ha, 6 locations were randomly zig-zag selected by placing a 0.13 m^2^ quadrat and ranking species by observed content as: 1 (70%), 2 (21%), or 3 (9%), totaling 48 locations monthly. All species presents were recorded even if not ranked 1, 2, or 3.

### Feedlot

Animals selected for GRAIN system were transported to UPREC in December of each year to acclimate to the system and were fed legume/grass baleage until the middle of February. Multiple phases of the ration were balanced in this period and fed to the animals to transition the diet from hay to the higher energy diet. Concentrate was slowly and incrementally added to their diet every 4–5 d until they were on their final feedlot high energy diet immediately before the trial began. Initially, 90% hay and 10% concentrate diet were supplied, and the amount of concentrate was increased until the ratio of hay:concentrate was 20:80 (DM base). Data from the adaptation period were excluded from the analysis. Nutrient composition of the ingredients used in the pre-trial period for each year is presented in Supplementary Table S1.

Steers were allocated to collective pens, providing at least 7 m^2^ per steer. Once daily, steers were fed a total mixed ration (TMR) formulated to contain 80% grain. The diet consisted of 20% orchardgrass hay, 74% corn, and 6% pellets. In YR1, the diet contained 50% dry corn and 24% high moisture corn (HMC), while in YR2 the diet contained 74% dry corn. Pellets contained 36% crude protein (N536, Kalmbach Feeds, INC. Upper Sandusky, OH).

Steers had ad libitum access to fresh water and free choice of mineral and vitamin block supplements (Prince Corporation, Marshfield, WI). The diet was offered once daily over 107 d in YR1 and 121 d in YR2. The ration was adjusted daily to maintain 3% to 5% refusals. The amount of feed offered and refusals were weighed per pen daily.

The TMR and orts were sampled weekly and stored at −20 °C. At the end of each month, samples from 2 wk were mixed according to group and a composited subsample was dried at 55 °C in a forced-air oven for 72 h, ground with a Wiley mill (1-mm screen; Arthur H. Thomas, Philadelphia, PA), and underwent subsequent chemical analysis.

### Feed Sample Collection and Analysis

All forage, TMR, and orts were separated for each group every 2 wk and analyzed for DM, ash, neutral detergent fiber (NDF), acid detergent fiber (ADF), crude protein (CP), and gross energy (GE). All nutrients were expressed as percentages of DM, determined by drying at 105 °C in a forced-air oven for at least 8 h. Ash content was determined after 6 h of oxidation at 500 °C in a muffle furnace. The NDF was analyzed according to Mertens (2002) with the inclusion of amylase and sodium sulfite. The ADF was analyzed according to AOAC (2000). Crude protein was determined according to Hach et al. (1987).

### Performance

A shrunk body weight (BW) was measured at the onset and end of the trial. A 12-h fasting body weight was recorded monthly and average daily gain (ADG) was determined via linear regression, and total gain was obtained by multiplying ADG by the number of days.

The length of time the animals were evaluated was determined based on forage mass availability, and all animals were slaughtered on the same date. The animals were slaughtered at the age of 18 and 26 mo (GRAIN and GRASS, respectively), at a commercial slaughter plant according to standard operating procedures.

### Carcass Traits

Final weight at slaughter was recorded the day before the slaughter. Hot carcass weight (HCW) was recorded for each animal. Dressing percent was calculated from the HCW divided by the final weight at slaughter and multiplied by 100. Carcass measurements were collected by a trained personnel 48-hr *postmortem* and included ribeye area (REA), 12th rib back fat, USDA yield grade, and marbling score.

A portion of the muscle longissimus lumborum (between 11th and 13th ribs) was collected from the left side of each carcass and the samples were transferred to the MSU Meat Laboratory in a cooler on ice packs within two hours. Two 2.54 cm-thick steaks were cut, individually vacuum packed and placed on stainless steel trays and aged at 4 °C for 14 d.

At day 14, one steak was frozen until the thawing and cooking loss analysis and Warner–Bratzler shear force (WBSF) analysis could be performed. The other fresh steak was evaluated for instrumental color and cooked for consumer panelists.

### Instrumental Surface Color

Color measurements were taken in three different locations on the surface of the meat using a Hunter MiniScan XE Plus (Model 4500L, aperture 25 mm, 45/0° illumination/viewing, illuminant D65, 10° standard observer, Hunter Labs, Inc. Reston, VA) colorimeter and averaged to represent the value for each steak. For each measurement, CIE lightness (L*), redness (a*), and yellowness (b*) color space values were recorded. The instrument was calibrated against a white and a black standard at the beginning of the measurement.

Hue angle and chroma values were calculated according to AMSA (2012). Chroma (color saturation) is a measure of the intensity of the red color, and it was calculated from the formula [(a*)^2^ + (b*)^2^}^0.5^ and hue angle, a measure of overall color, was calculated from arc-tan b*/a*.

### Sensory Attributes

A sensory analysis protocol was approved by the Michigan State University Institutional Review Board before this study (Study number #1799). In YR1, the consumer test was performed at the testing area in the Department of Food Science, MSU, East Lansing, MI. In YR2, it was performed at Matrix Sciences Company located in Grand Rapids, MI due to COVID restrictions in place at MSU at the time. The steaks were always transferred in coolers on ice packs and the tests were performed by the same people in both years.

For each year, the steaks were cooked for consumer panelists (*n* = 105) to evaluate flavor, texture, juiciness, and overall acceptability using a 9-point hedonic scale (1 = dislike extremely and 9 = like extremely). A quality attribute panel was used to evaluate the samples.

The study consisted of 15 groups of seven people each year. Each group tested the same steaks, one from each of the four treatments. The steaks were selected randomly, unpacked, and the subcutaneous fat was trimmed off. The steak was cooked to an internal temperature of 71 °C using a clamshell electric grill (George Foreman) that was preheated. After cooking, the steaks were allowed to rest at room temperature for three min. The steaks were cut into 1.27 × 1.27 × 2.54 cm pieces, and all external fat and connective tissue was removed.

The cubes from each treatment were identified with random three-digit-codes and placed in an individual covered container and served to each panelist. Panelists were given four containers with each treatment (each sample was represented by two pieces) at the same time, beside one cup filled with distilled water and unsalted saltine crackers. Panelists were first asked to take a bite of cracker and a sip of water to cleanse their palate before starting, and between each sample.

### Water Holding Capacity

A 2.54 cm-thick steak was used for thawing and cooking loss and shear force analyses. The samples were stored at −24 °C under vacuum for approximately 35 d before the analyses. The steaks were thawed at a temperature of 4 °C for 24 h before cooking.

The steaks were then weighed and cooked to an internal temperature of 71 °C using a clamshell electric grill (George Foreman) that was preheated for at least 15 min. The cooked samples rested at room temperature for five min and the weight was recorded. Thawing and cooking loss were expressed as the percentage of weight loss against fresh weight.

### Warner–Bratzler Shear Force

The cooked steaks were cooled down overnight at 4 °C. Six to eight 1.27-cm diameter cores were obtained from each steak parallel to the muscle fibers (AMSA, 2015) using a drill press mounted corer. The cores were subjected to shear force measurement using a TA-XT Texture Analyzer (Stable Micro System Ltd., UK) fitted with a V-shaped Warner–Bratzler blade. Samples were cut through the slit of the table as the blade moved down with a constant speed of 20 cm/min. Each core was sheared once, so that the blade cut across the muscle fiber. The mean of the cores was utilized for statistical analysis.

### Statistical Analysis

All data were analyzed using the GLIMMIX procedure in SAS (SAS 2014 Institute Inc., Cary, NC, v 9.4). The distribution of model residual was tested for normality and homogeneity using Shapiro-Wilk and Cochran tests. Backfat, USDA yield grade, thawing loss, cooking loss, WBSF, color measurements, and sensory attributes were log transformed.

Data for growth performance, carcass traits, and meat quality traits were analyzed using the fixed effect of genotype, finishing system, year, and their interaction. Sensory panel results were also analyzed using a randomized complete design in SAS, and the sensory panelist was the random effect. Depending on the variable, the individual steer, carcass, or steak were considered as the experimental unit. The three-way interaction was not significant, and it was removed from the model.

The PDIFF option of the LSMEANS statement was used when comparing treatment means. Statistical differences were considered significant when the *P*-value was less than or equal to 0.05, and tendencies were considered when the *P*-value was greater than 0.05 and less than 0.10. Lower and upper confidence limits at 95% were reported for each treatment.

## RESULTS

### Weather Conditions and Diet Quality

The total rainfall observed throughout the experimental period was 470.4 mm in YR1 and 645.2 mm in YR2 (Figure 1A). Daily mean air temperature increased from the beginning of the trial until July and then decreased over time in each year. The maximum temperature ranged from 27.1 to 8.9 °C and the minimum temperature ranged from 14.1 to −1.1°C.

**Figure 2.**
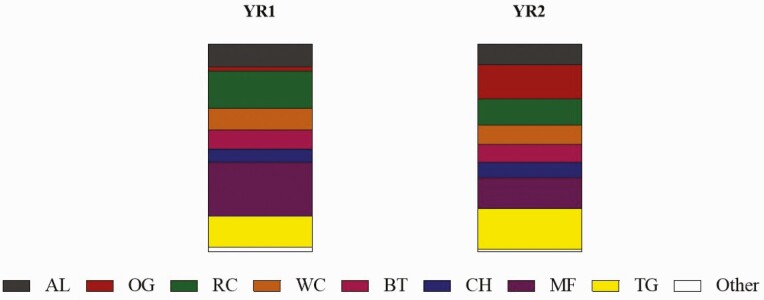
Pasture botanical composition in 2019 (YR1) and 2020 (YR2) in percentage of dry matter. AF = Alfalfa, OG = Orchardgrass, RC = Red Clover, WC = White Clover, BT = Birdsfoot Trefoil, CH = Chicory, MF = Meadow Fescue, TG = Timothy grass.

Pre-graze forage mass averaged 4,298.4 and 4,372.4 kg of DM and post-graze averaged 2,702.1 and 2,695.9 kg of DM in YR1 and YR2, respectively (Figure 1B). Post-graze forage mass corresponded to 62.9% in YR1 and 61.7% in YR2 of the pre-graze mass. Botanical composition for each year is detailed in Figure 2. A variation was observed between the years as expected. In YR1, the pasture was dominated by meadow fescue at 25.9%, followed by red clover (18.0%), timothy grass (15.0%), alfalfa (11.0%), and white clover (10.4%). Each other species accounted for less than 10%, together totaling 19.7%. In YR2, timothy grass (19.8%) was the dominant species and orchard grass (16.6%) was the second most present species, followed by meadow fescue (15.0%), and red clover (12.7%). The other species combined totaled 35.9%. Meadow fescue and red clover dropped from YR1 to YR2, while orchard grass and timothy grass increased considerably.

Forage and diet nutritive values for each year are presented in Table 1. As expected, pre-graze forage was higher in quality, including greater CP and reduced values for both NDF and ADF, compared to post-graze. Crude protein increased from 11.5 to 15.0% in DM basis from YR1 to YR2. The TMR and orts had consistent values between years, except for DM. The lower DM value observed in YR1 is due to the inclusion of HMC (24% of the DM) in the diet compared to no HMC in YR2. The averaged values for both years were 9.7% crude protein, 20.3% NDF, and 9.7% ADF.

**Table 1.** Nutritive value of forage (pre and post graze) and feedlot diets (TMR and orts) in 2019 (YR1) and 2020 (YR2)

Item % DM	YR1				YR2			
	Pre	Post	TMR	Orts	Pre	Post	TMR	Orts
Dry matter, %	20.5	26.9	75.9	74.6	22.1	25.3	85.4	78.9
Ash	7.1	6.7	4.4	4.8	6.1	5.8	3.0	4.3
Crude protein	11.5	8.7	9.7	9.6	15.0	12.3	9.6	8.9
NDF^1^	52.2	59.1	21.2	20.6	51.5	55.5	19.9	32.1
ADF^2^	35.0	38.5	10.0	9.8	32.0	34.0	9.4	16.6
TDN^3^	62.0	59.1	78.9	79.2	62.3	60.6	79.6	72.7

^1^NDF = Neutral detergent fiber.

^2^ADF = Acid detergent fiber.

^3^TDN = Total digestible nutrients. The TDN was estimated using the formula recommended by Capelle et al. (2001): TDN (%) = 83.790 − 0.4171 × NDF (forage) and TDN (%) = 91.0246 − 0.571588 × NDF (TMR and orts).

### Performance, Carcass Traits, and Meat Quality

The main effects (beef genotype, finishing system, and year) for all variables evaluated are presented in Tables 2 and 3. When any of the two-way interactions were significant, the data were presented as a graph. *P*-values for all variables, including the main effects and their interactions, are presented in Supplementary Table S2.

**Table 2.** Effects of beef genotypes and finishing system on performance and carcass traits (mean and lower and upper confidence limit at 95%)

Item	Genotype^1^		System		Year	
	RA	AK	GRASS	GRAIN	2019	2020
Growth^2^						
Initial BW, kg	454.2^(445.6 − 462.9)^	459.7^(451.1 − 468.4)^	**436.5** ^(428.7 − 444.2)^	**477.5** ^(467.8 − 487.2)^	454.4^(446.7 − 462.2)^	459.5^(449.9 − 469.2)^
Final BW, kg	592.8^(581.9 − 603.6)^	590.6^(579.8 − 601.5)^	**563.5** ^553.8 − 573.2)^	**619.9** ^(607.7 − 632.1)^	**579.9** ^(570.2 − 589.6)^	**603.5** ^(591.3 − 615.6)^
Total gain, kg	134.6^(129.6 − 139.6)^	128.5^(123.5 − 133.5)^	**119.4** ^(115.0 − 123.9)^	**143.7** ^(138.1 − 149.3)^	**120.5** ^(116.0 − 125.0)^	**142.6** ^(137.0 − 148.2)^
ADG, kg d^−1^	1.25^(1.21 − 1.31)^	1.20^(1.15 − 1.25)^	**1.19** ^(1.15 − 1.24)^	**1.26** ^(1.21 − 1.32)^	**1.28** ^(1.24 − 1.32)^	**1.18** ^(1.12 − 1.23)^
Carcass^3^						
Wgt at slaughter, kg	571.6^(561.3 − 582.0)^	572.3^(561.7 − 582.9)^	**547.4** ^(538.2 − 556.7)^	**596.5** ^(584.6 − 608.4)^	**559.7** ^(550.5 − 569.0)^	**584.2** ^(572.3 − 596.1)^
HCW, kg	337.4^(331.2 − 343.7)^	345.7^(339.4 − 351.9)^	**314.9** ^(309.3 − 320.5)^	**368.2** ^(361.2 − 375.2)^	**333.2** ^(327.6 − 338.8)^	**349.9** ^(342.8 − 356.9)^
Dressing, %	**59.1** ^(58.7 − 59.4)^	**60.5** ^(60.1 − 60.9)^	**57.6** ^(57.3 − 57.9)^	**61.9** ^(61.5 − 62.4)^	**59.5** ^(59.1 − 59.8)^	**60.1** ^(59.7 − 60.5)^
Backfat, mm	9.7^(8.9 − 10.7)^	9.9^(9.0 − 10.9)^	**5.9** ^(5.5 − 6.4)^	**16.3** ^(14.7 − 18.1)^	9.6^(8.8 − 10.4)^	10.1^(9.1 − 11.2)^
Ribeye area, cm^2^	**72.1** ^(70.2 − 74.0)^	**74.9** ^(73.0 − 76.8)^	**69.2** ^(67.5 − 70.9)^	**77.8** ^(75.7 − 80.0)^	72.3^(70.6 − 74.0)^	74.7^(72.6 − 76.9)^
Ribeye, cm^2^ CW^−1^	9.7 ^(9.4 − 9.9)^	9.9 ^(9.6 − 10.1)^	9.9 ^(9.7 − 10.2)^	9.6 ^(9.3 − 9.9)^	9.9 ^(9.6 – 10.1)^	9.7 ^(9.4–10.0)^
USDA yield grade	2.9^(2.8 − 3.0)^	2.9^(2.7 − 3.0)^	**2.4** ^(2.3 − 2.5)^	**3.5** ^(3.3 − 3.6)^	2.9^(2.8 − 3.1)^	2.9^(2.7 − 3.0)^
Marbling Score	**494.5** ^(474.8 − 514.3)^	**529.2** ^(509.5 − 549.0)^	**404.5** ^(386.9 − 422.1)^	**619.2** ^(597.1 − 641.3)^	519.2^(501.5 − 536.8)^	504.6^(482.5 − 526.7)^

^1^RA = Red Angus; AK = Red Angus x Akaushi crossbred

^2^BW = Body weight; ADG = Average daily gain

^3^HCW = Hot carcass weight; Marbling scores: Choice− = 400–499, Choice0 = 500–599, Choice+ = 600–699.

Bold values indicate that the main effect was statistically different (*P* < 0.05). *P*-values are shown in Supplementary Table S2.

**Table 3.** Effects of beef genotypes and finishing system on meat quality and sensory attributes (mean and lower and upper confidence limit at 95%)

Item	Genotype^1^		System		Year	
	RA	AK	GRASS	GRAIN	2019	2020
Water holding capacity						
Thawing loss, %	1.0^(0.9 − 1.1)^	1.0^(0.9 − 1.0)^	**1.1** ^(1.0 − 1.2)^	**0.9** ^(0.8 − 1.0)^	**0.8** ^(0.8 − 0.9)^	**1.2** ^(1.1 − 1.4)^
Cooking loss, %	22.7^(21.7 − 23.8)^	23.2^(22.1 − 24.2)^	22.4^(21.5 − 23.2)^	23.6^(22.4 − 24.8)^	22.4^(21.5 − 23.3)^	23.5^(22.3 − 24.7)^
Color						
Lightness (L*)	33.2^(32.3 − 34.1)^	33.6^(32.7 − 34.5)^	**29.4** ^(28.7 − 30.1)^	**37.9** ^(36.7 − 39.0)^	**36.0** ^(35.1 − 36.9)^	**30.9** ^(30.0 − 31.9)^
Redness (a*)	21.0^(20.4 − 21.7)^	21.1^(20.5 − 21.8)^	**23.2** ^(22.5 − 23.8)^	**19.1** ^(18.5 − 19.8)^	**17.8** ^(17.3 − 18.3)^	**25.0** ^(24.1 − 25.9)^
Yellowness (b*)	21.1^(20.6 − 21.5)^	21.0^(20.6 − 21.5)^	**21.5** ^(21.1 − 21.9)^	**20.6** ^(20.1 − 21.1)^	**17.3** ^(17.0 − 17.6)^	**25.6** ^(25.0 − 26.2)^
Hue angle	44.9^(44.4 − 45.4)^	44.8^(44.3 − 45.4)^	**42.8** ^(42.3 − 43.2)^	**47.1** ^(46.4 − 47.7)^	**44.1** ^(43.6 − 44.6)^	**45.7** ^(45.2 − 46.3)^
Chroma	29.9^(29.2 − 30.6)^	29.9^(29.1 − 30.6)^	**31.6** ^(31.0 − 32.3)^	**28.2** ^(27.4 − 29.0)^	**24.9** ^(24.3 − 25.4)^	**35.9** ^(34.9 − 36.9)^
Shear force^2^						
WBSF, kg	4.1^(3.8 − 4.3)^	4.2^(4.0 − 4.5)^	**4.6** ^(4.4 − 4.8)^	**3.7** ^(3.5 − 4.0)^	**3.7** ^(3.5 − 3.9)^	**4.6** ^(4.3 − 5.0)^
Sensory^3^						
Flavor—liking	5.9 ^(5.6 − 6.1)^	5.8^(5.6 − 6.1)^	**5.5** ^(5.3 − 5.7)^	**6.2** ^(6.0 − 6.5)^	6.0 ^(5.7 − 6.3)^	5.7 ^(5.4 − 6.0)^
Juiciness—liking	5.5^(5.3 − 5.8)^	5.8^(5.6 − 6.1)^	**5.4** ^(5.1 − 5.6)^	**6.0** ^(5.7 − 6.2)^	5.6 ^(5.3 − 5.9)^	5.7 ^(5.4 − 6.0)^
Texture/firmness—liking	6.0 ^(5.8 − 6.3)^	5.9^(5.7 − 6.1)^	**5.5** ^(5.3 − 5.7)^	**6.4** ^(6.2 − 6.7)^	6.0^(5.7 − 6.2)^	5.9^(5.7 − 6.2)^
Overall acceptability	5.9^(5.6 − 6.1)^	5.8^(5.6 − 6.0)^	**5.4** ^(5.1 − 5.6)^	**6.3** ^(6.1- 6.6)^	6.0 ^(5.7 − 6.3)^	5.7 ^(5.4 − 6.0)^

^1^RA = Red Angus; AK = Red Angus x Akaushi crossbred.

^2^WBSF = Warner–Bratzler Shear Force.

^3^Sensory: Panelists assigned steak attributes using 9-point scales (1 = dislike extremely; 9 = like extremely) for flavor, juiciness, texture/firmness, and overall acceptability.

Bold values indicate that the main effect was statistically different (*P* < 0.05). *P*-values are shown in Supplementary Table S2.

### Performance

**Figure 3.**
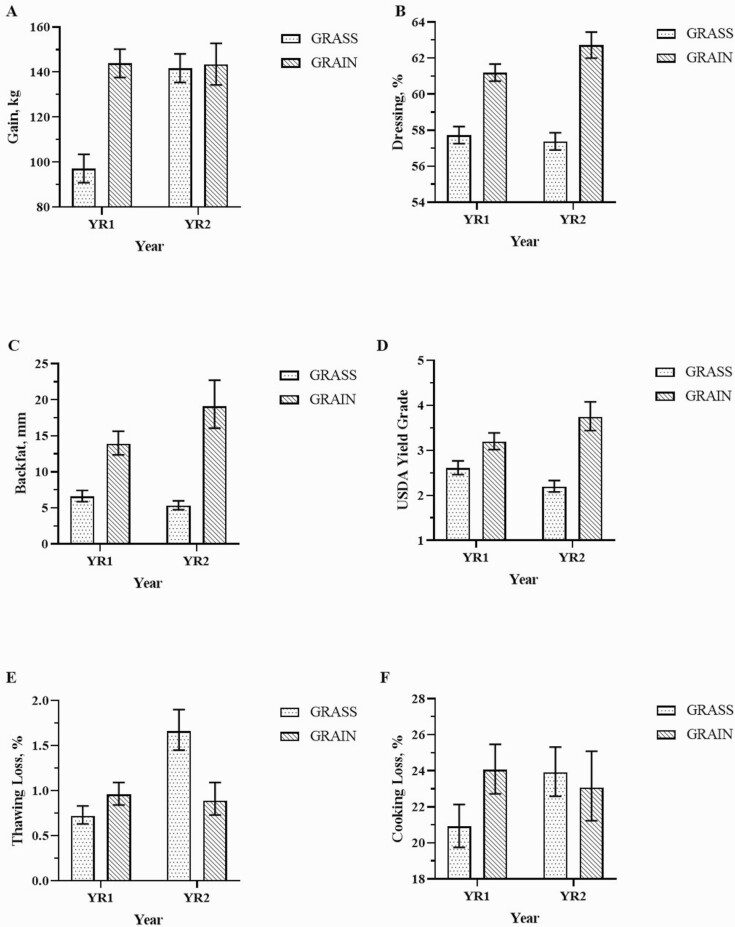
Animal gain, carcass traits, and water holding capacity of the two-way interaction between finishing system and year. (A) Total gain, (B) dressing percent, (C) backfat, (D) USDA yield grade, (E) thawing loss, and (F) cooking loss. Bars indicate mean values and error bars indicate upper and lower confidence limits.

Both initial and final BW were significantly impacted by finishing system (*P* < 0.01), but neither were impacted by genotype. Initial and final BW were greater for GRAIN than for the GRASS system (*P* < 0.01). Final BW was also impacted by year; steers in YR2 had greater BW compared to YR1 (603.5 vs. 579.9 kg, *P* < 0.01). Total gain was significantly impacted by system and year (*P* < 0.01) and the results mirrored those of final BW. There was a tendency (*P* = 0.09) for greater gain in RA than AK steers (134.6 vs. 128.5, *P* = 0.09). A system by year interaction was significant for total gain (Figure 3A, *P* < 0.01). In YR1, steers from GRASS had a lower total gain compared to those in GRAIN (97.1 vs. 141.7 kg), and no difference was observed in YR2. There were both system and year significant effects for ADG. Steers finished under the GRAIN system had greater ADG compared to those under the GRASS (1.26 vs. 1.19 kg d^−1^, *P* = 0.04), and ADG was greater in YR1 than YR2 (*P* < 0.01).

### Carcass Traits

All carcass traits were impacted by finishing system (*P* < 0.01). Steers in GRAIN had the greatest values for all variables evaluated compared to those in GRASS, except that GRASS had a tendency (*P* = 0.06) for higher ribeye area by carcass weight than GRAIN. There was a genotype effect for dressing (*P* < 0.01) and marbling score (*P* = 0.01), where AK steers had the greatest values (60.5% and 529 vs. 59.1% and 494 for AK and RA, respectively). There was a tendency for the AK steers to have greater HCW (345.6 kg, *P* = 0.06) and ribeye area (74.9 cm^2^, *P* = 0.06) compared to RA (337.4 kg and 72.1 cm^2^). Year was significant for weight at slaughter (*P* < 0.01), HCW (*P* < 0.01), dressing (*P* = 0.01), and ribeye area (*P* = 0.08), with the greatest values observed in YR2.

**Figure 4.**
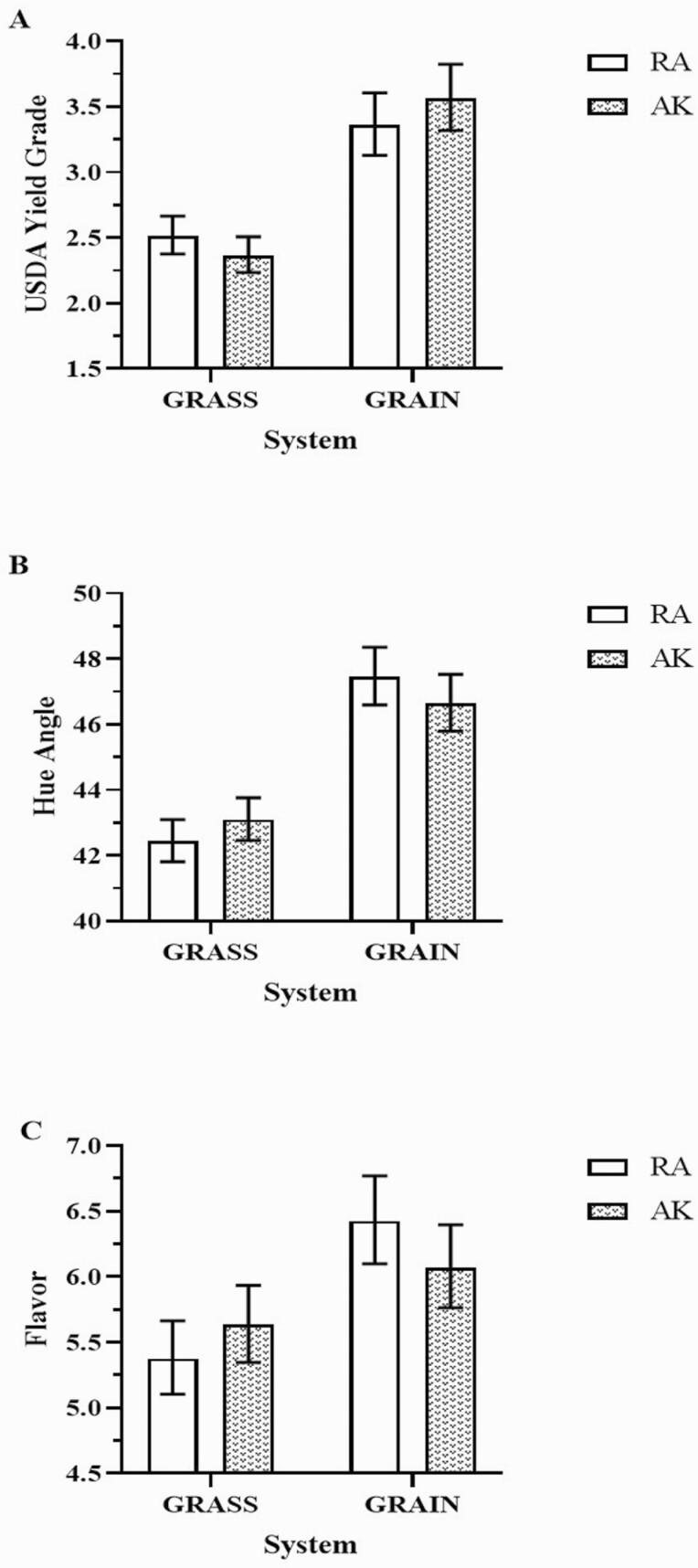
Two-way interaction between beef genotype and finishing system for (A) USDA yield grade, (B) hue angle, and (C) flavor. Bars indicate mean values and error bars indicate upper and lower confidence limits. RA = Red Angus; AK = Red Angus x Akaushi crossbreed.

There was a system by year interaction for dressing, backfat, and USDA yield grade (Figure 3B–D, *P* < 0.01). USDA also had a genotype by system interaction (Figure 4A, *P* = 0.06) and no genotype by year interaction was observed for any of the carcass traits. Dressing was lower in GRASS than GRAIN in both years. No difference was observed in GRASS between the years, but for GRAIN dressing was lower in YR1 than in YR2 (Figure 3B). The same pattern was observed for backfat, with lower values in GRASS compared to GRAIN in both years. No difference was observed for GRASS between YR1 and YR2, but for GRAIN, backfat was lower in YR1 than in YR2 (Figure 3C). USDA yield grade was lower in GRASS than GRAIN in both years. For GRASS, steers from YR1 had a greater USDA yield grade than those from YR2, and for GRAIN, YR1 had the lowest value (Figure 3D). Regarding the genotype by system interaction, USDA yield grade was lower in GRASS compared to GRAIN in both genotypes, and no difference was observed between the two genotypes for any GRASS or GRAIN systems (Figure 4A).

### Meat Quality

There was no difference in water holding capacity for genotypes (Table 3, *P* > 0.05), but thawing loss was lower in GRAIN than GRASS (0.9 vs. 1.1%, *P* = 0.03), and it was greater in YR2 than YR1 (1.2 vs. 0.8%, *P* < 0.01). There was a system by year interaction for both thawing and cooking loss (*P* < 0.01). Thawing loss was lower in GRASS than GRAIN in YR1 but was greater in GRASS than GRAIN in YR2. No difference was observed for GRAIN between YR1 and YR2, but for GRASS it was lowest in YR1 (Figure 3E). Cooking loss was lower in GRASS than GRAIN in YR1, and no difference was observed between systems in YR2. For GRASS, cooking loss was greater in YR2 than YR1, and for GRAIN, no difference was observed between years (Figure 3F).

**Figure 5.**
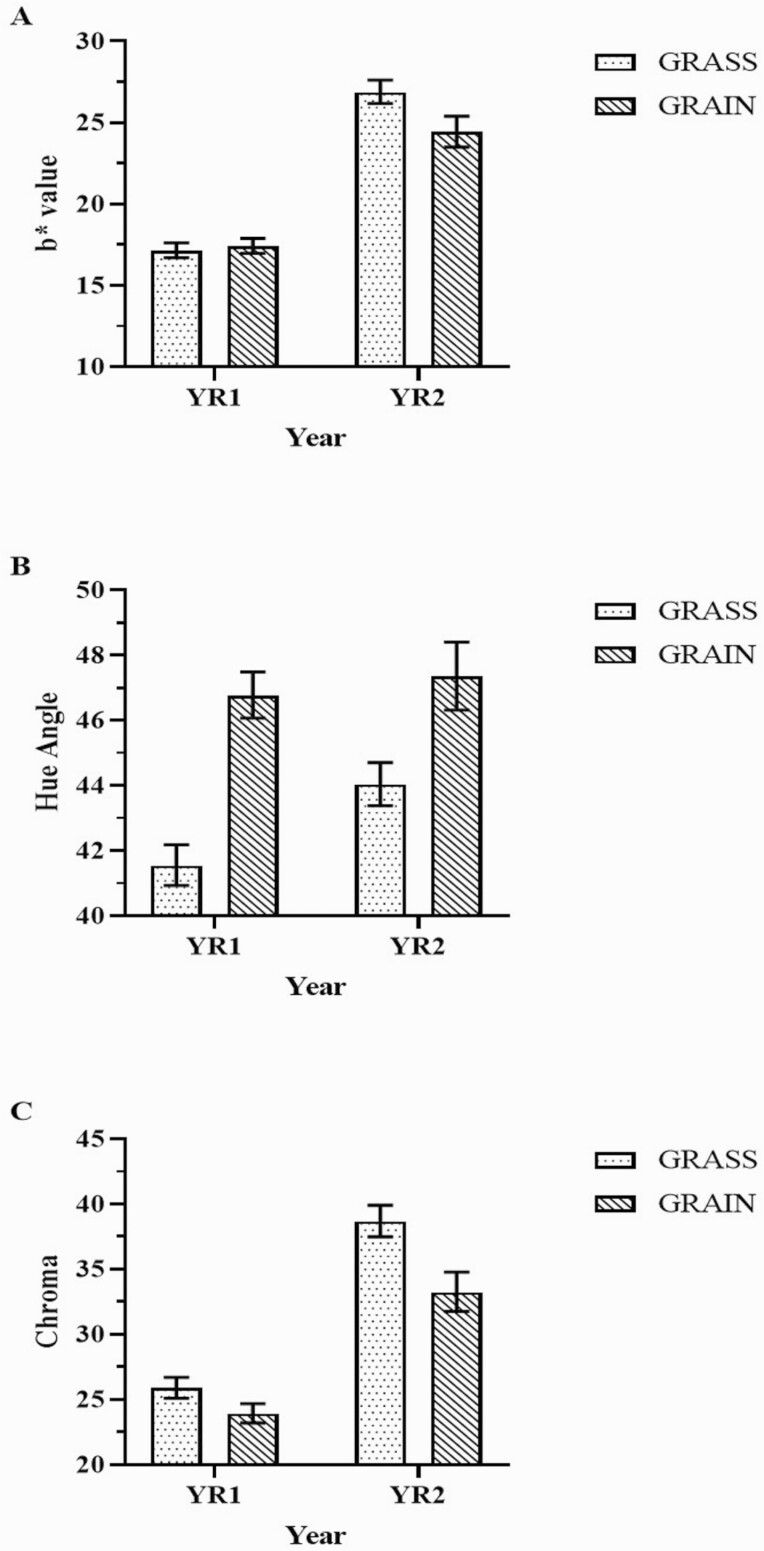
Color measurements of the two-way interaction between finishing system and year for b* value (A), hue angle (B), and chroma (C). Bars indicate mean values and error bars indicate upper and lower confidence limits.

Color variables were impacted by system and year (Table 3, *P* < 0.01). L* color parameter (lightness) and hue angle presented greater values while a* (redness), b* (yellowness), and chroma presented lower values in GRAIN compared to GRASS. Regarding the year effect, L* had the greatest value while all other variables had the lowest values in YR1. A significant interaction between system and year was observed for b*(*P* < 0.01), hue angle (*P* < 0.01), and chroma (*P* = 0.04). No difference was observed for b* between GRASS and GRAIN in YR1, but GRAIN had a lower value compared to GRASS in YR2. Also, both GRASS and GRAIN presented lower values in YR1 compared to YR2 (Figure 5A). For hue angle, GRASS presented lower values compared to GRAIN in both years (*P* < 0.01). For GRASS, YR1 presented a lower value than YR2; however, for GRAIN no difference was observed between the years (Figure 5B). For chroma, GRAIN presented the lowest values in both years, and for both systems, YR1 presented a lower value than YR2 (Figure 5C).

### Sensory Attributes and Shear Force

All sensory attributes were significantly impacted by system (Table 3, *P* < 0.01), but no effects of genotypes were observed, except a tendency for juiciness (Supplementary Table S2, *P* = 0.06). The attributes were scored greater in GRAIN than GRASS beef. Steaks from AK tended to be juicier than RA (5.8 vs. 5.5). There was a genotype by system interaction for flavor (*P* = 0.02). Beef from RA had lower flavor liking in GRASS than in GRAIN, and no difference was observed for AK. No difference was observed for flavor between RA and AK within each system (Figure 4C).

There were both system and year effects for WBSF (*P* < 0.01), no difference for genotype (*P* = 0.51) nor any interaction. Beef from steers in GRASS had greater WBSF than those from GRAIN (*P* < 0.01). Regarding the year effect on WBSF, YR2 presented greater values compared to YR1 (*P* < 0.01).

## DISCUSSION

### Performance

Results of this study demonstrate that the finishing system has greater impact on animal growth, carcass traits, and meat quality of beef cattle compared to cattle genotype. The quality of the diet was the main factor that influenced these differences. Although the crude protein of the diet was greater in GRASS, forages usually present an unbalanced protein: energy ratio. This is mainly because the energy content is low in forages (approximately 61% compared to 79% in TMR), which is the main limitation to achieving optimal animal performance in grazing diets (Orjales et al., 2019; Teobaldo et al., 2020). The efficiency of energy utilization decreases with high CP due to the energy cost of urea synthesis from excessive ammonia in the liver (NRC, 2016). Although the quality of forage is an important factor for crude protein levels, the species cultivated also change the nutritional value of the diet consumed by animals on pasture. For example, pastures containing clover are rich in N whereas CP levels of perennial ryegrass are lower than that of the leguminous species (Van Vuuren and Dasselaar, 2006; Orjales et al., 2019). Our pasture was rich in legume, since it contained more than 20% of clover and 10% of alfalfa (Figure 2), which explain the high values of CP observed.

Initial BW differences for steers in the two finishing systems may be attributed to the pre-trial management. Even though GRASS steers were 6 mo older than those in GRAIN, the steers assigned to GRAIN were fed in a feedlot system around 7 mo before the beginning of the trial each year, while the steers assigned to GRASS grazed for the whole period. Therefore, steers from GRASS were lighter than those from GRAIN. The difference between days in trial between GRASS and GRAIN impacted the total gain and ADG. GRASS steers remained in the finishing system 28 d less than those in GRAIN in YR1. The low forage quantity observed in that year was a consequence of low precipitation (Figure 1), especially at the beginning of the growing season. As a result, the lower total gain and final BW observed in YR1 can be attributed to the shorter trial duration for GRASS in that particular year.

Variations in both total gain (20%) and ADG (6%) were more in animals in GRAIN than animals in the GRASS system. Asizua et al. (2017) showed that regardless of the duration of finishing and genotype, the growth rates of bulls in the feedlot system were almost twice the growth rate of animals grazing natural pastures. However, the mixed forage provided in the current study led to a comparable ADG between the steers in both systems, which demonstrates that pasture management can result in a considerably higher growth rate of animals in grazing systems.

The only difference in growth observed between the genotypes was a tendency for higher total gain in RA compared to AK steers. Radunz et al. (2009) compared Angus and Wagyu sired steers on feedlot and reported a greater ADG and DMI in Angus cattle. However, Wagyu had improved feed efficiency compared to Angus cattle.

### Carcass Traits

Finishing system had by far the biggest effects on carcass traits. As expected, animals from the GRAIN system had a greater weight at slaughter, which explains the higher values observed for HCW, dressing, backfat, and ribeye area when comparing both finishing systems. In addition, the high-energy diet changed the average marbling score from USDA low choice to high choice. The energy content of the diet for the GRAIN system resulted in the rapid growth of the cattle and thereby a greater deposition of intramuscular fat (Wood et al., 2008, Bautista-Martínez et al., 2020). Similar results were also reported by Alfaia et al. (2009). Besides the low level of energy for the GRASS diet, higher energy requirements for physical activity when grazing could also explain the lower carcass fat (NRC, 2016; Hamdi et al., 2016).

Differences in carcass traits between the genotype groups were as expected. Studies have reported variation in beef fat deposition due to genetic differences between cattle breeds (Mwangi et al., 2019). Despite no difference for weight at slaughter between the genotype groups, carcass weight was heavier for AK, with a greater ribeye area and marbling score, as expected. Radunz et al. (2009) also reported greater both ribeye area and marbling scores for Wagyu carcasses compared to Angus.

The importance of genotypes in increasing marbling scores for beef production is clear and well known. Wagyu or Akaushi genetically influenced cattle have been well characterized to deposit more intramuscular and less subcutaneous fat (Xie et al., 1996; Mir et al., 1999; Wheeler et al., 2004). We expected that RA x AK crossbreds would improve quality grade in grazing systems, our results have shown that AK had greater marbling score than RA, but there was no interaction between beef genotypes and finishing system.

### Meat Quality

The water lost during fresh meat storage contains vitamins, minerals, and flavor compounds (Rodriguez-Estrada et al., 1997; Gerber et al., 2009). Water holding capacity varies mainly with carcass final pH and post-mortem proteolysis (Huff-Lonergan and Lonergan, 2005). Hamdi et al. (2016) observed that despite the unchanged pH, drip loss in raw meat was higher for lambs fed in feedlot compared to those in pasture, and for cooked meat, water holding capacity was not altered by the feeding system. Macit et al. (2003) observed that vitamin E supplementation in a concentrate and grass hay diet for lambs reduced lipid oxidation and drip loss in the meat. Our results showed different patterns between years; thawing loss in grass-fed steaks was lower in YR1 and higher in YR2 compared to grain-fed. Cooking loss was not impacted by the main factors. Similar results were found by Acciaro et al. (2021), where a pasture-based treatment decreased the intramuscular fat of the meat without affecting the cooking loss. Fruet et al. (2018) also did not find difference for cooking loss between beef from grass or grain finishing systems. However, our results suggest that, for cooked steaks, water holding capacity was greater for GRASS in the first year. Differences observed for cooking loss can be related to more intramuscular fat in GRAIN than GRASS steak in our study.

Changes in muscle color suggested an association between lipid oxidation, vitamin E concentration, and color (Warren et al., 2008b; Li and Liu, 2012; Burnett et al., 2020). The fatty acid composition of both the diets and beef from this study has been evaluated and will be presented in another manuscript. Oxidation of some fatty acids, such as α-Linoleic (ALA) or eicosapentaenoic (EPA) and docosahexaenoic (DHA) acid, can cause oxidation of the myoglobin pigment leading to the formation of metmyoglobin on the surface of beef products (Jacobsen, 2008). When measured objectively, this leads to reduced a* values, increased calculated surface myoglobin percentage, and increased hue angle, or color saturation (chroma) in meat products (Burnett et al., 2020).

According to Warren et al. (2008b), the intensity of the red color, i.e., chroma, declined gradually as the display period progressed (from 24 h to 7 d after packaging) but the decline was faster in the muscles of the concentrate-fed groups than in the grass silage-fed ones. Also, the authors observed a lower intensity of red color in the concentrate group, which agrees with the results found in the current study. The feeding system can affect the lipid composition and vitamin E concentration in the meat (Bekhit et al., 2013). Generally, grass-based-diets are rich in α-tocopherol and β-carotene (Descalzo and Sancho, 2008; Daley et al., 2010; Acciaro et al., 2021), which prevents excess oxidation of the fatty acids in meat products and slows down the conversion of deoxymyoglobin and oxymyoglobin to metmyoglobin (Wood and Enser, 1997). The addition of vitamin E to feedlot diets produced beef with reduced hue angle (less metmyoglobin) (Juárez et al., 2012) and increased a* values (Phelps et al., 2017).

The results found by Acciaro et al. (2021) showed that the pasture-based treatment decreased the intramuscular fat and increased the a-tocopherol concentration of meat without affecting the color parameters. On the other hand, Warren et al. (2008b) compared the grazing pasture-fed and grain-fed cattle and found that antioxidants naturally present in the pasture probably caused higher levels of vitamin E, lower lipid oxidation, and better color retention in the meat.

Furthermore, the influence of feeding systems on meat color is related to glycogen content and muscle pH (Jorquera-Chavez et al., 2019; Santos et al., 2021). Grass-fed animals tend to have a darker meat color with higher pigmentation than grain-fed ones, which could be related to the higher physical activity in pasture systems resulting in a higher oxidative muscle capacity (Priolo et al., 2001). In addition, forage-based diets have lower glycogen content, generating lower acidification of the muscles and consequently a darker color during *post-mortem* (Mancini, 2009). Similar to our results, other studies comparing concentrate-based and pasture-based systems have shown that the latter resulted in lower L* and higher a* (Legako et al., 2018; Mezgebo et al., 2019).

### Sensory Attributes

There were significant and consistent effects of finishing systems on sensory quality. Beef sensory attributes can be impacted by many factors. Feeding regimes can alter the rate and extent of proteolysis and consequently influence beef tenderness (Gagaoua et al., 2019; Wicks et al., 2019). While Bruce et al. (1991) found that steers fed high energy diets produce carcasses with increased tenderness, Resconi et al. (2010) and Del Campo et al. (2008) found that the increase in the energy content of the diet decreased both tenderness and flavor intensity of grass-fed beef when compared with more intensive feeding systems. It has been hypothesized that the greater vitamin E content in meat of pasture-fed cattle increases the collagen (Purslow et al., 2012), which improves meat tenderness.

Fatty acid composition of adipose tissue also affects its firmness. According to Wood (1984), this happens because the different fatty acids have different melting points, between approximately 25–50 °C, with saturated fatty acids melting at higher temperatures and polyunsaturated fatty acids at lower temperatures for example C18:0 melts at 69 °C and C18:2*n*-6 at 5 °C. Ekine-Dzivenu et al. (2017) found genetic correlations between fatty acids in beef tissue and meat tenderness, suggesting that they are likely influenced by a subset of the same genes in beef cattle. The results of fatty acid composition in the current study will be evaluated and presented later; thus, a correlation between the fatty acid composition and the sensory quality scores will be evaluated.

The results observed for WBSF matched with the texture in the sensory test. No difference was observed between the genotypes, but beef from steers in GRASS had higher shear force than those from GRAIN. Some authors have shown that the texture of beef from animals reared in a grazing system can be more tender than that of the beef from concentrate-fed animals (Bruce et al., 2004; Realini et al., 2004), which may have been due to differences in pH or both carcass pH and temperature decline post-mortem. The higher carcass weight and fatness of animals in GRAIN may have slowed the decline in carcass internal temperature which might have reduced tenderness. Texture also varied according to the sex of the cattle. Berger et al. (2018) evaluated the effect of the aging method after 28 d and reported shear force values varying from 3.1 to 3.3 kg in steaks from grass-fed heifers. Fruet et al. (2018) observed that beef from steers finished on grass was more cohesive than beef finished on grain (4.9 vs. 4.7 kg), but the variation in texture was not correlated with negative effects on objective and subjective tenderness for pasture-finishing meat.

The results for the two genotypes were quite similar and there was no evidence of a decrease in texture or an increase in beef flavor in AK as expected as a consequence of the greater marbling score. However, a tendency for a higher juiciness score was observed for steaks from AK. The total lipid content of muscle such as intramuscular fat has a role in the tenderness and juiciness of cooked meat although the strength of the correlation varies considerably between studies (Wood et al., 2008). According to the current results, the marbling fat may be related to the difference observed for juiciness between RA and AK.

Finishing systems were a more important factor than beef genotype affecting sensory quality and shear force. Several US studies have found that grain-fed cattle generally rank higher than grass-fed cattle for the main attributes of beef tenderness and flavor (Schroeder et al., 1980; Medeiros et al., 1987). The low sensory scores in grass-fed cattle can be linked to light carcasses and low-fat levels. Although the growth rate in the finishing phase was similar between grass- and grain-fed cattle, animals in GRASS had slower growth before the trial and it may have delayed the beginning of the fat deposition. According to Bidner et al. (1986) and Muir et al. (1998) when the growth rate is similar between systems, diet effects on sensory attributes are much smaller. Besides the growth rates, other factors also play an important role in the sensory profile, such as the carcass weight, fat levels, marbling score, among others (Warren et al., 2008a; French et al., 2003).

## CONCLUSIONS

Results of this study have shown that beef finishing system has a significant impact on animal performance, carcass traits, meat quality and sensory attributes of the meat. The grain finishing system not only produced heavier carcasses with greater marbling scores than the grass finishing system, but also had lower shear force and a marked impact on steaks’ sensory attributes and consumer acceptability. In addition, the color attributes were influenced by the finishing system, and the results were more favorable for the grass-fed meat.

The AK had greater dressing percentage and ribeye area compared to RA steers, as well as a higher marbling score. Both genotypes had similar sensory scores, with only small indications that AK might produce meat with higher beef juiciness than RA, which likely may be related to high marbling.

## Supplementary Material

txab214_suppl_Supplementary_Tables_S1-S2
