# Supplementary material for: Influence of beef genotypes on animal performance, carcass traits, meat quality, and sensory characteristics in grazing or feedlot-finished steers
Source: Transl Anim Sci. 2021 Sep 21;5(4):txab214. doi: 10.1093/tas/txab214 (PMC8651173; doi:10.1093/tas/txab214)
Supplement: txab214_suppl_Supplementary_Tables_S1-S2 [file txab214_suppl_supplementary_tables_s1-s2.docx]

**Supplementary material**

**Table S1**. Nutrient composition of the ingredients used in the pre-trial period for the GRAIN treatment.

| **Nutrient Composition,**  **% DM** | **YR1** | | |  | **YR2** | | |
| --- | --- | --- | --- | --- | --- | --- | --- |
|  | **Hay** | **Dry corn** | **Pellet^2^** |  | **Hay** | **Dry corn** | **Pellet^3^** |
| Dry matter, % | 76.7 | 81.1 | 85.0 |  | 81.7 | 85.5 | 86.4 |
| Ash | 8.0 | 1.6 | 20.8 |  | 5.3 | 1.2 | 20.6 |
| Crude Protein | 9.9 | 5.8 | 34.6 |  | 7.2 | 7.4 | 35.4 |
| NDF^1^ | 63.4 | 9.3 | 10.8 |  | 65.1 | 10.2 | 10.0 |
| ADF^2^ | 36.2 | 3.1 | 5.6 |  | 38.7 | 3.2 | 5.4 |

^1^NDF = Neutral detergent fiber

^2^ADF = Acid detergent fiber

Pellet^3^ (N536, Kalmbach Feeds, INC. Upper Sandusky, Ohio)

| Variables |  | *P*-values | | | | | | |
| --- | --- | --- | --- | --- | --- | --- | --- | --- |
|  | Genotype^1^  (G) | | System  (S) | Year  (Y) | G*S | G*Y | S*Y | G*S*Y |
| *Growth*^2^ |  | |  |  |  |  |  |  |
| Initial BW, kg | 0.37 | | <0.01 | 0.42 | 0.53 | 0.54 | 0.10 | 0.84 |
| Final BW, kg | 0.78 | | <0.01 | <0.01 | 0.81 | 0.94 | 0.45 | 0.90 |
| Total gain, kg | 0.09 | | <0.01 | <0.01 | 0.42 | 0.17 | <0.01 | 0.89 |
| ADG, kg d^-1^ | 0.15 | | 0.04 | <0.01 | 0.45 | 0.24 | 0.11 | 0.96 |
| *Carcass*^3^ |  | |  |  |  |  |  |  |
| Weight at slaughter, kg | 0.92 | | <0.01 | <0.01 | 0.66 | 0.91 | 0.69 | 0.74 |
| HCW, kg | 0.06 | | <0.01 | <0.01 | 0.47 | 0.83 | 0.52 | 0.68 |
| Dressing, % | <0.01 | | <0.01 | 0.01 | 0.11 | 0.95 | <0.01 | 0.23 |
| Backfat, mm | 0.80 | | <0.01 | 0.45 | 0.29 | 0.16 | <0.01 | 0.90 |
| Ribeye area, cm^2^ | 0.04 | | <0.01 | 0.08 | 0.91 | 0.33 | 0.17 | 0.32 |
| Ribeye, cm^2^ CW^-1^ | 0.33 | | 0.06 | 0.39 | 0.54 | 0.45 | 0.27 | 0.54 |
| USDA yield grade | 0.95 | | <0.01 | 0.85 | 0.06 | 0.20 | <0.01 | 0.94 |
| Marbling Score | 0.01 | | <0.01 | 0.31 | 0.37 | 0.10 | 0.46 | 0.31 |
| *Water capacity* |  | |  |  |  |  |  |  |
| Thawing loss, % | 0.55 | | 0.03 | <0.01 | 0.76 | 0.34 | <0.01 | 0.23 |
| Cooking loss, % | 0.57 | | 0.11 | 0.16 | 0.50 | 0.36 | <0.01 | 0.40 |
| *Color* |  | |  |  |  |  |  |  |
| Lightness (L*) | 0.55 | | <0.01 | <0.01 | 0.33 | 0.41 | 0.13 | 0.11 |
| Redness (a*) | 0.86 | | <0.01 | <0.01 | 0.31 | 0.39 | 0.35 | 0.46 |
| Yellowness (b*) | 0.96 | | <0.01 | <0.01 | 0.75 | 0.52 | <0.01 | 0.97 |
| Hue angle | 0.91 | | <0.01 | <0.01 | 0.05 | 0.50 | <0.01 | 0.15 |
| Chroma | 0.96 | | <0.01 | <0.01 | 0.65 | 0.43 | 0.04 | 0.64 |
| *Shear Force* |  | |  |  |  |  |  |  |
| WBSF^4^, kg | 0.51 | | <0.01 | <0.01 | 0.43 | 0.56 | 0.37 | 0.45 |
| *Sensory*^5^ |  | |  |  |  |  |  |  |
| Flavor | 0.83 | | <0.01 | 0.12 | 0.02 | 0.84 | 0.18 | 0.97 |
| Juiciness | 0.06 | | <0.01 | 0.67 | 0.19 | 0.99 | 0.61 | 0.57 |
| Texture/firmness | 0.49 | | <0.01 | 0.93 | 0.35 | 0.51 | 0.89 | 0.84 |
| Overall acceptability | 0.75 | | <0.01 | 0.14 | 0.31 | 0.30 | 0.48 | 0.77 |

**Table S2**. *P*-values for the main factors (Beef genotype, finishing system and year) and their interaction for all variables evaluated

^1^RA = Red Angus; AK = Red Angus x Akaushi crossbreed

^2^BW = Body weight; ADG = Average daily gain

^3^HCW = Hot carcass weight; Marbling scores: Choice- = 400-499, Choice0 = 500-599, Choice+ = 600-699.

^4^WBSF = Warner-Bratzler Shear Force;

^5^Sensory: Panelists assigned steak attributes using 9-point scales (1 = dislike extremely; 9 = like extremely) for ﬂavor, juiciness, texture/firmness, and overall acceptability.
